# Microrheology of DNA hydrogel gelling and melting on cooling†
†Electronic supplementary information (ESI) available. See DOI: 10.1039/c8sm00751a


**DOI:** 10.1039/c8sm00751a

**Published:** 2018-06-19

**Authors:** Javier Fernandez-Castanon, Silvio Bianchi, Filippo Saglimbeni, Roberto Di Leonardo, Francesco Sciortino

**Affiliations:** a Dipartimento di Fisica , “Sapienza” Università di Roma , Rome , I-00185 , Italy . Email: javier.fernandez.castanon@roma1.infn.it; b CNR-NANOTEC , Soft and Living Matter Laboratory , Rome , I-00185 , Italy; c CNR-ISC , UOS “Sapienza” Università di Roma , Rome , I-00186 , Italy

## Abstract

We present systematic characterisation by means of dynamic light scattering and particle tracking techniques of the viscosity and of the linear viscoelastic moduli, *G*′(*ω*) and *G*′′(*ω*), for two different DNA hydrogels. These thermoreversible systems are composed of tetravalent DNA-made nanostars whose sticky sequence is designed to provide controlled interparticle bonding. While the first system forms a gel on cooling, the second one has been programmed to behave as a re-entrant gel, turning again to a fluid solution at low temperature. The frequency-dependent viscous and storage moduli and the viscosity reveal the different viscoelastic behavior of the two DNA hydrogels. Our results show how little variations in the design of the DNA sequences allow tuning of the mechanical response of these biocompatible all-DNA materials.

## Abstract 2

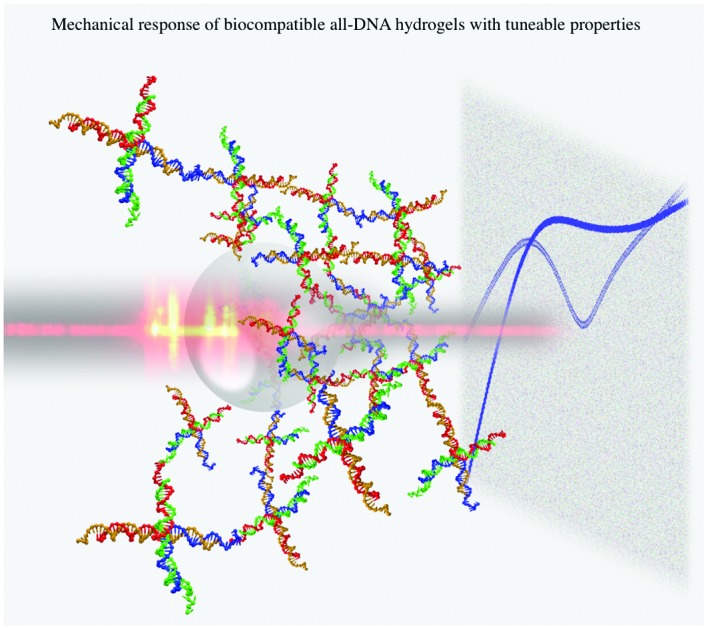
Mechanical response of biocompatible all-DNA hydrogels with tuneable properties.

## 1. Introduction

DNA nanotechnology, a fast growing field originating from the insightful vision of N. C. Seeman,^1^ has expanded the interest in DNA beyond genetics and biology. Over the last decades, DNA has acquired a remarkable role as a molecular building block at the nanoscale.^2^,^3^ Several protocols and techniques have been developed for the design of complex 2D^4^,^5^ and 3D^6^–^8^ geometries and networks. The binding specificity and programmability of the DNA nucleotides, together with the continuous reduction in the costs of production of synthetic DNA, permit design of DNA sequences programmed to self-assemble in bulk quantities of desired nanostructures with controlled binding properties and tuneable response to external phenomena such as pH,^9^ temperature,^10^ or light.^11^,^12^ Along these lines, beside being thoroughly investigated in single-molecule studies,^13^–^15^ DNA has been also successfully applied to investigate the kinetic control of oil droplet functionalized colloids,^11^ to drive the assembly of nanoparticles into macroscopic materials,^16^ to organize nanocrystal molecules,^17^ and to guide the crystallization of colloidal nanoparticles.^18^ In addition, the hydrophilic character of DNA is exploited for the formation of 3D networks^19^,^20^ able to hold large amounts of water, similar to polymeric hydrogels,^21^ a property of major interest for the development of drug delivery, sensing, and tissue engineering applications.^20^

**Fig. 1:**
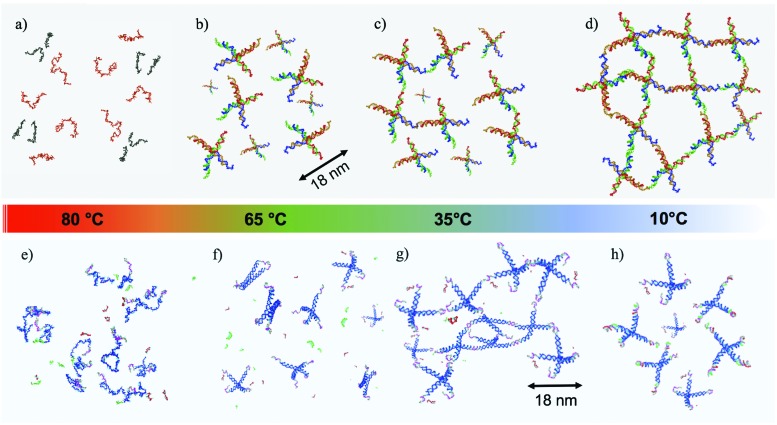
Self-assembly process, from high to low temperatures, both for the NS (a–d) and the RG (e–h) systems. (a and e) represent the very high temperature case, where all DNA sequences are not hybridized, remaining single-stranded in the buffer solution. When *T* is lowered down to ∼65 °C, the four strands (designed to self-assemble in tetrafunctional units both in the NS (b) and in the RG (f) systems) are now hybridised. In the RG case, the competitor sequences (in green and red colors) are still in solution in a non-hybridised state, being characterized by a lower melting temperature. At intermediate *T* ∼ 35 °C the NSs start to bind to each other *via* complementary sticky ends (c and g), resulting in both cases in the formation of a connected network. In the RG case the competitor sequences are still in single strand form. Finally, at low *T* the two systems behave differently. In the NS case (d) NSs form a fully bonded network with very long living inter-particle bonds (thus mimicking the behavior of a chemical gel). In the RG system, the competitors have displaced the bonds that were previously forming the network, creating a solution of freely diffusing particles.

Most of the literature on rheological properties of DNA solutions has focused on long single and double stranded DNA, interpreted with models of entangled semiflexible polymer coils.^22^–^24^ Experimental characterisation of the mechanical properties of materials created starting from purposefully designed DNA-particles is highly welcome and, only very recently, a microrheology study of three-functional Y-DNA particles has been submitted.^25^ A novel experimental microfluidic-based active microrheology approach to investigate how designed three-functional DNA-hydrogels evolve from diffusive liquid-like systems to shear-thinning fluids on cooling, passing through a series of intermediate power-law fluid states has recently been published.^26^ Here, we focus on the rheological properties of two DNA hydrogels composed of tetrafunctional DNA nanostars (NSs in the following). In both cases, the NSs are assembled from four properly designed DNA single-strands of about forty bases. At the end of each of the four arms, a short self-complementary single-strand sequence provides a well characterised binding overhang, commonly indicated as a sticky end. Below the melting temperature of the sticky end sequence, the particles bind in a three-dimensional tetravalent network in which each NS acts as a network site. The first NS system is a well characterised all-DNA sample that progressively gels on cooling.^10^,^27^ The second system has been designed to display re-entrant gelation^28^ (the RG system in the following). As for the NS system, the RG system evolves from a fluid at high temperature to a well developed highly connected network in which all DNA NSs are bonded on cooling. This gel then transforms to a fluid phase on further cooling, a behavior that is achieved due to the additional presence in solution of short DNA sequences acting as competitors. Exploiting competitive interactions, these short DNA sequences, at low temperature, contend with the sticky sequences completely capping the NS overhangs. As a result, the system melts both at high and at low temperature.^28^,^29^ The self-assembling pathways for both systems as a function of temperature (*T*) are depicted in Fig. 1.

We report dynamic light scattering (DLS) and particle tracking microrheology (PTM) experiments to quantify the mechanical properties of these two DNA hydrogels in a frequency (*ω*) domain ranging from 1 rad s^–1^ to 10^5^ rad s^–1^ and their viscosities (*η*). DLS provides an easy-to-implement experimental set-up in which only the size and concentration of the colloidal tracers has to be carefully selected. PTM provides measurements of the sample *η* by tracking over long times, thus significantly extending the DLS range, the relative position between two colloids embedded in the same field of view. These two methods result in valuable information about the mechanical properties of these materials and about their flow and deformation properties as functions of *ω* at different *T*.

## 2. Materials and methods

### 2.1. DNA hydrogel preparation

Both examined samples are based on tetravalent networks whose nodes are provided by tetrafunctional DNA-NSs. In the NS case, the tetrafunctional DNA NSs interact directly *via* a self-complementary sticky sequence of six bases. In the case of the RG gel the tetrafunctional A particles are bonded *via* a self-complementary sticky sequence of eight bases. These eight bases are both preceded and followed by three extra bases which act as toeholds easing the attachment of the competitor B sequences. A detailed discussion of the base sequences for both systems can be found in the original articles^28^,^30^ and in Section II of the ESI.†

Samples at tetrafunctional unit concentrations of 248 μM (the NS system) and 142 μM (the RG system) in H_2_O 100 mM NaCl buffer have been prepared. To grasp the meaning of these concentrations, these values should be compared with the values required to form a fully bonded network, respectively of ∼220 μM (the NS system^30^) and ∼110 μM (the RG system^28^). Thus, both systems lie well within the equilibrium gel^31^ concentration window. Samples were slowly annealed at high *T* to guarantee the proper self-assembly of the tetrafunctional units before the formation of the gel and sealed in 2.4 mm inner diameter borosilicate glass capillaries. Detailed observation with a video camera equipped with a 100× magnification lens confirmed that phase separation occurred for none of the investigated samples. One sample was prepared for each of the systems to evaluate their dynamic transition throughout the selected range of *T*.

### 2.2. DLS microrheology

To perform passive microrheology measurements we add to the DNA solution polystyrene (PS) colloids (530 nm in size) coated with streptavidin from Sigma-Aldrich at a volume fraction *φ* = 3.4 × 10^–4^. As discussed in Section I of the ESI,† this concentration is sufficiently small to prevent colloid–colloid interactions and at the same sufficiently large to provide a scattering signal significantly larger than the one generated by the DNA particles. In this way, the measured scattered intensity provides essential information on the self-motion of the PS tracer particles. DLS measurements were performed with a He–Ne *λ* = 633 nm laser at a fixed angle *θ* = 90°. Temperatures were controlled by a programmable thermostat and an independent thermometer in contact with the sample bath, allowing for 40 minutes of thermalisation prior to 40 min long measurements. A Brookhaven correlator provides the intensity autocorrelation functions *g*_2_(*t*) ∼ ) ∼ 〈*I*(*q*,*t*)*I*(*q*,0),0)〉 where where *q* is the wave vector. The electric field correlation function *g*_1_(*t*) is then evaluated exploiting the Siegert relationship.^32^ The well-established constrained regulation method for continuous distributions^33^ (CONTIN) was used to provide a well-behaved representation of the *g*_1_(*t*) correlation functions *via* a sum of exponential decays. The viscoelastic moduli *G*′(*ω*) and *G*′′(*ω*) are then evaluated as described in detail in Section IV of the ESI.†

### 2.3. PTM microrheology

DLS experiments access the time window 1 μs–10 s. With particles of size ∼530 nm, viscosities lower than a few Pa s are accessed. At higher values of *η*, the measured *g*_1_(*t*) relaxes at times larger than the maximum experimental accessible correlation time thwarting the acquisition of accurate measurements, especially in the *T* region in which a highly connected network of long-lived bonds is formed. To access this larger *η* window we performed tracking measurements with PTM.

We used a custom-built microscope equipped with a 100× Nikon objective (1.4 numerical aperture). For each *T*, bright field images were acquired over a temporal window of 10 min at frame-rates ranging from 10 to 50 fps. In the bright field, a 530 nm PS microsphere gives a strong intensity peak in the image when the focal plane is positioned slightly below it. We isolate the intensity peak applying a threshold to the image. Subsequently the 2D coordinates of the microsphere can be extracted by applying a “center of mass” tracking algorithm.^34^,^35^ Due to the high viscosity of the medium, the microspheres' displacements were limited (from ∼1 μm at the highest *T*, down to ∼20 nm at the lowest *T*). In order to reach an accurate measurement of the diffusion coefficient it was crucial to eliminate inevitable drifts and vibrations of the setup. These were ruled out by considering the relative position between two microspheres that were found in the same field of view. At all the investigated *T*, the same two particles were tracked, and the relative position vector coordinates between the two microspheres undergo a diffusive motion with an effective diffusion coefficient given by the sum of the single particles' diffusion coefficients. The diffusion coefficient was extracted from a linear fit of the measured mean square displacement (MSD), and subsequently the viscosity was calculated by means of the Stokes–Einstein relation (eqn (S17) of the ESI†). Hydrodynamic interactions between the two microspheres were neglected since the correction to the diffusion coefficient for the selected particle sizes and distances (>7 μm) is expected to be below 1%.^36^ Such a correction is smaller than the error introduced by the particles' radial polydispersity which is of the order of ∼5%.

## 3. Results and discussion

DLS microrheology lies within the class of passive microrheological techniques in which no stress is externally applied on the probes but just their passive motion originating from the thermal fluctuations is measured.^37^,^38^ Specifically, we observe the thermally driven motion of a tracer particle added in solution (a streptavidin coated polystyrene (PS) particle) by measuring the time correlation of the scattered light, *e.g.* the electric field autocorrelation function *g*_1_(*t*) as a function of time *t*, and at a fixed scattering angle *θ* = 90°. Our experimental set-up and capillary sample cuvette do not allow us to investigate the *q*-dependence. We provide proof of a diffusive motion of the tracer particles by comparing the DLS results with PTM techniques (see Section 3.3 of the main text and Section III of the ESI† for details) which by measuring trajectories over long-times allows us to directly visualize the colloidal diffusive motion. In addition, the good agreement of the results from independent measurements and experimental techniques supports the assumption of diffusive motion of the probe colloids and of the absence of *q*-dependent effects.

### 3.1. DNA nanostar hydrogel

**Fig. 2:**
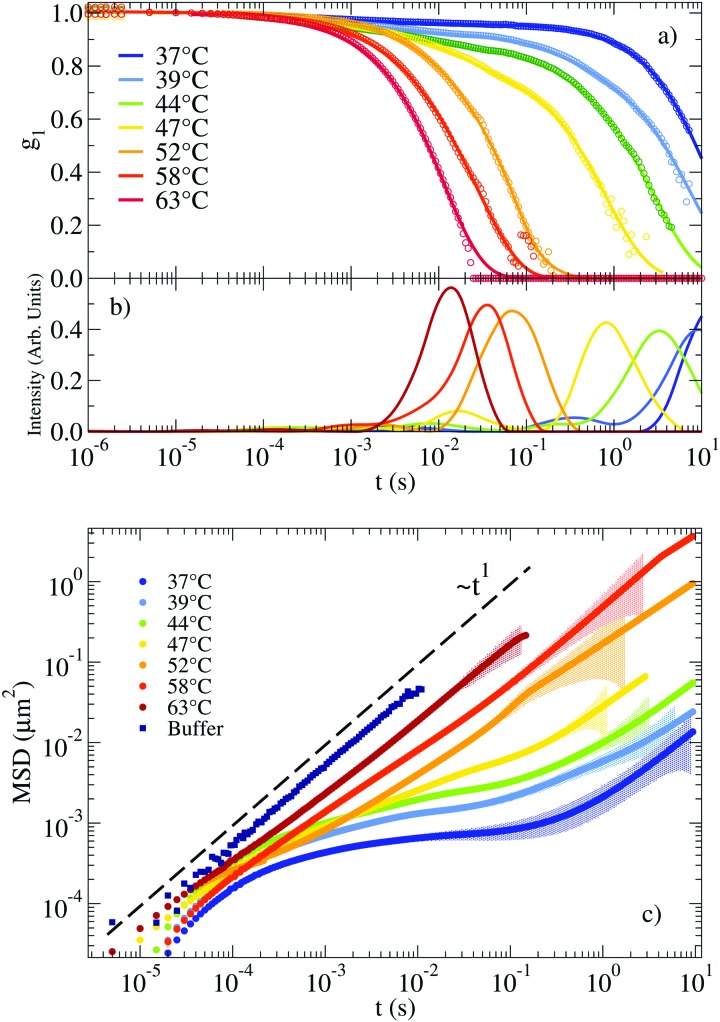
(a) Autocorrelation functions measuring the motion of a probe particle immersed in a NS solution at a concentration of 248 μM. Experimental data is represented by circles. Solid lines indicate the result of applying CONTIN to the experimental *g*_1_(*t*). (b) Corresponding distribution of relaxation times as calculated from CONTIN. (c) Corresponding MSD of the probe particles (symbols). The coloured shadow backgrounds represent the statistical error calculated following Section V of the ESI.† The errors are not shown for values ΔMSD(*t*)/MSD(*t*) > 1. The figure also shows for comparison the MSD of the same tracers dispersed in a buffer of H_2_O and 100 mM NaCl (see Section I of the ESI†).

The autocorrelation functions *g*_1_(*t*) for the NS system are displayed in Fig. 2(a) in the range of *T* between 63 °C and 37 °C. For lower *T*, the system relaxes at times beyond the largest experimentally accessible time (10 s). The experimental data are superimposed on the CONTIN^33^ regularization results, which provide a smooth representation of the measured correlation functions. Fig. 2(b) shows the corresponding CONTIN distribution of relaxation times for each *T*. The MSD of the tracer particles, calculated as discussed in Section IV of the ESI,† are shown in Fig. 2(c). The three sets of equivalent data manifest the change in dynamics on cooling. At high *T* the correlation function decays with a single characteristic time (a single-peak in the CONTIN distribution) and the MSD displays a linear time dependence, consistent with a pure diffusive motion of the tracer particles and viscous behaviour typically associated with Newtonian fluids. This single-peak profile constitutes a hallmark of one single source of scattering and it is consistent with the 20 times larger scattering intensity of the solution with probe colloids compared to the pure DNA–water system. On progressively cooling, the formation of the bonds between distinct NSs slows down the tracers dynamics and deviations from the viscous-like response appear, consistent with the gradual emergence of elastic forces originating from the progressive formation of the viscoelastic network. At low *T* the tracer particles become trapped in the gel network for times comparable to the bond lifetime. In these conditions, the correlation function develops clear two-step relaxation with two different amplitudes, the CONTIN distribution of relaxation times displays two-peak behaviour and correspondingly, a clear plateau emerges in the MSD, whose value indicates the amplitude of the tracer motion in the cage. The amplitude of the slow relaxation process, now different from one, and the corresponding plateau in the MSD indicate the predominant elastic nature of the NS hydrogel (see the following discussion of *G*′(*ω*) and *G*′′(*ω*)) on the time scale of the NS–NS bond lifetime.

**Fig. 3:**
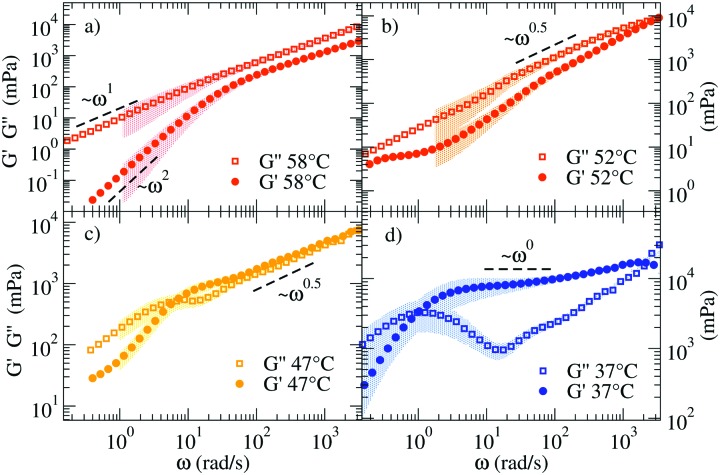
Loss (*G*′′) and storage (*G*′) moduli of the NS system for (a) 58 °C, (b) 52 °C, (c) 47 °C, and (d) 37 °C. The coloured shadows indicate the error bars calculated according to Section V in ESI.† Error bars are displayed only for Δ*G*′/*G*′ < 1 and Δ*G*′′/*G*′′ < 1.

Fig. 3 shows the linear viscoelastic moduli at four representative *T*, calculated according to the procedure described in the Methods section. At the highest *T* (58 °C) the viscous component *G*′′(*ω*) of the complex modulus G*(*ω*) takes higher values than those of the elastic modulus *G*′(*ω*) showing the typical *ω* and *ω*^2^ scaling at low frequency and growth consistent with a power-law with exponent 0.75 ± 0.03 at large *ω*. Such a power-law exponent is close to the value predicted for polymers by the Zimm model (in *Θ* solvent) *ω*^2/3^.^39^ In fact, at these *T* all NSs are independently floating in the solution and thus the viscous behavior of the material is expected to be predominant. On cooling (see 47 °C and 52 °C), *G*′(*ω*) and *G*′′(*ω*) start to develop more complex functional dependence. At 47 °C both moduli are characterised by parallel power-law growth ∼*ω*^*n*^ at large frequencies with *n* ≈ 0.47, signatures of the proximity to a percolation point.

While the power-law dependence is a clear indication of a system approaching the percolation threshold, the transformation of *n* into a structural quantity such as the fractal dimension *d*_f_ requires some approximations. In previous work,^40^ the value of *n* has been found to vary significantly depending on the network properties, clustering around the value *n* = 0.5.^40^–^43^ Factors like stoichiometry, chain length and dilution of the critical gels play a role in the composition and structure of the systems and therefore significant variations in the *n* values have been reported.^41^ To name a few, in the case in which excluded volume interactions can be neglected and hydrodynamic interactions are fully screened, theory predicts^42^–^44^ that *G*′(*ω*) = *G*′′(*ω*) ∼ *ω*^2/(*d*_f_+2)^. For the three-dimensional percolation universality class, *d*_f_ = 2.5 and *G*′(*ω*) = *G*′′(*ω*) ∼ *ω*^0.44[combining macron]^. We observe a similarity in the response of the two moduli over a limited frequency range. Different from chemical gels where the self-similar response of moduli occurs over a wide frequency range and terminal flow is not observed, here the finite lifetime of the bonds between NSs introduces a lower-frequency crossover as observed in Fig. 3(c).

At 37 °C (Fig. 3(d)) the system is well within percolation. *G*′(*ω*) becomes dominant over *G*′′(*ω*) while *G*′′(*ω*) shows a minimum at *ω* ≈ 20 rad s^–1^, suggesting that the diffusive motion of the colloids is arrested on the corresponding timescale. In addition, *G*′(*ω*) and *G*′′(*ω*) cross over at *ω*_c_ ∼ 1 rad s^–1^. This crossover frequency reflects the inverse of the relaxation time of the system and it conventionally indicates the transition from a fluid-like (viscous) to a rubbery, solid-like (elastic) frequency region. For comparison, at 47 °C, the crossover is found at a higher *ω*, in accordance with a faster relaxation time of the probe colloid (see Fig. 2(a)) for a weaker network stabilised at higher *T*. In all the investigated samples, at *ω* < *ω*_c_ terminal fluid behavior is recovered with *G*′(*ω*) ∼ *ω*^2^ and *G*′′(*ω*) ∼ *ω*^1^.

When a clear plateau in *G*′(*ω*) is present, its value can be used to provide an estimate of the cage size *ξ* confining the probe particle (of radius *a*), according to the relation in eqn (S15) of the ESI.† We observe plateau values of *G*′(*ω*) up to 7 Pa, corresponding to *ξ* ≈ 26 nm. As a reference, we note that the order of magnitude of the elastic modulus can be estimated by the product of the density of elastically effective chains times the thermal energy.^45^,^46^ In the fully bonded low *T* limit, each NS contributes to two effective chains, suggesting as the (DLS experimentally unaccessible) low *T* limiting value *G*′(*ω*_plateau_) ≈ 600 Pa. Unfortunately, as commented before, at such low *T g*_1_(*t*) would decorrelate at much longer times than the maximum accessible experimental time of DLS techniques.

### 3.2. Re-entrant hydrogel

The RG system^28^ was originally proposed as a proof of concept of the possibility to design DNA constructs mimicking peculiar colloidal particles at the nanoscale. Specifically, it exploits the competition between different bonding patterns resulting in a system of tetravalent DNA particles that can be either a fluid state of diffusive clusters or a spanning tetravalent network *i.e.* a highly viscous gel (see Fig. 1). Interestingly the gel phase is designed to be sandwiched between two fluid phases. On cooling, the system exhibits a cross-over from fluid to solid to fluid again, providing a theoretical example of a material that can be hardened on heating. Here we explore the viscoelastic properties of this novel material to reveal this double cross-over in a sequence of states that evolves from fluid to percolation to fully bonded gel to percolation and to fluid again.

**Fig. 4:**
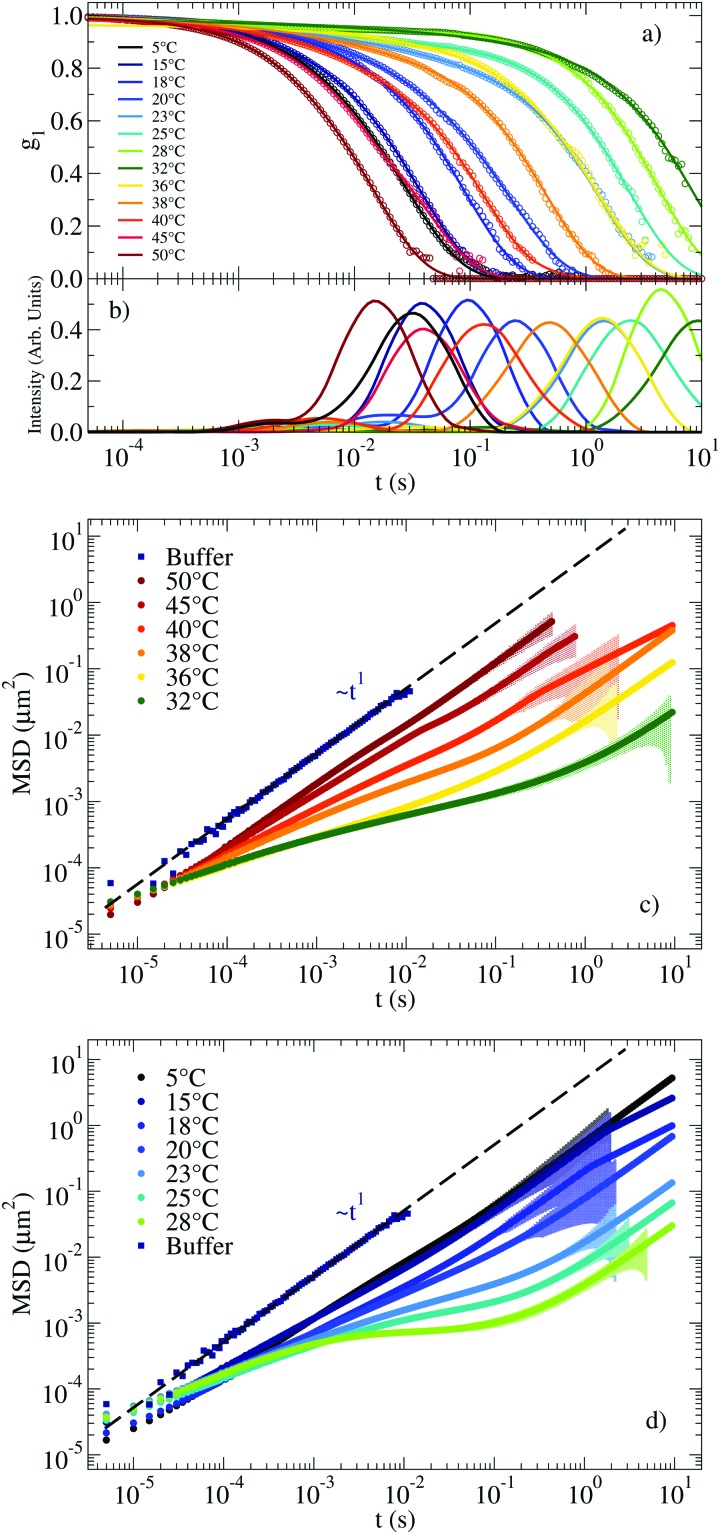
(a) *g*_1_(*t*) measuring the motion of a probe particle dissolved in the RG hydrogel at a NS concentration of 110 μM. Experimental data is represented by circles and the result of applying CONTIN procedures is indicated with solid lines. (b) Corresponding distribution of relaxation times as calculated from CONTIN. (c) MSD for *T* ranging between 50 °C and 32 °C and (d) for *T* ranging between 28 °C and 5 °C. The coloured shadow backgrounds represent the statistical error calculated following Section V of the ESI.† The errors are not shown for values ΔMSD(*t*) > MSD(*t*).

The corresponding experimental *g*_1_(*t*) for the RG system are displayed in Fig. 4(a) together with their CONTIN analysis. The *g*_1_(*t*) functions clearly show an increase in the relaxation times on cooling from 50 °C to 32 °C. On further lowering *T* the relaxation times become faster and faster, recovering a typical fluid-like value at the lowest investigated *T*. This behavior is replicated in the associated MSD curves in Fig. 4(c) for the high *T* regime (where the dynamics gets slower on cooling) and in Fig. 4(d) for low *T* (where the dynamics gets faster on cooling). Thus, the viscous high and low *T* behavior is comprised (see the MSD at *T* ≈ 30 °C) of the caging regime typical of non linear viscoelastic systems, similar to the one observed in the NS system at low *T* (see Fig. 2(c)).

**Fig. 5:**
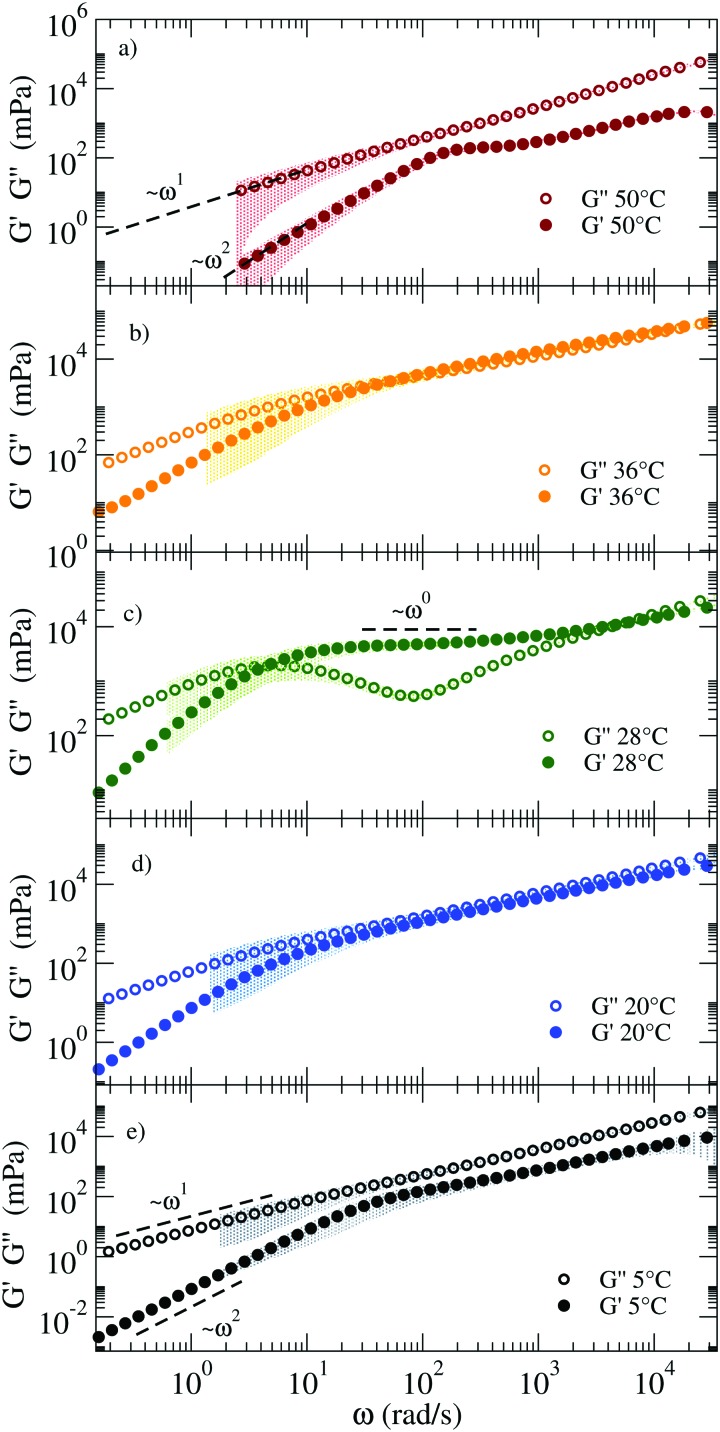
*G*′ and *G*′′ moduli of the RG system from high (top) to low (bottom) *T*, showing mechanical features characteristic of different states: (a) a fluid at 50 °C, (b) close to the percolation threshold at 36 °C, (c) a viscoelastic solid at 28 °C, again (d) close to percolation at 20 °C and (e) a fluid at 5 °C. The coloured shadows indicate the error bars calculated according to Section V in ESI.†

Fig. 5 shows the corresponding viscoelastic moduli for different characteristic *T*. The data clearly show on cooling the viscoelastic behavior typical of a fluid (a), of a percolating system (b), of an elastic gel (c), again of a percolating system (d) and of a low *T* fluid (e). Indeed, panels (a) and (e) are characterised by *G*′′(*ω*) > *G*′(*ω*) in such a way that the loss modulus prevails over the elastic modulus in the absence of crossovers in the whole *ω* range. Panels (b) and (d) are characterised by *G*′′(*ω*) ≈ *G*′(*ω*) for *ω* > 10^2^ rad s^–1^ and by clear power-law frequency dependences with a scaling exponent *n* again around 0.5 (best fit values of *n* = 0.46 and *n* = 0.58 respectively). Finally, panel (c) shows the typical behavior of a viscoelastic solid where *G*′′(*ω*) shows a minimum at *ω* ≈ 500 rad s^–1^ and a corresponding plateau value of *G*′(*ω*) of about 8 Pa. We associate this behavior with an elastic response where the colloidal particle is trapped in a cage. It must be noted that our DNA hydrogels are physical gels in which the bond breaking and formation events, absent in chemical gels, are responsible for the final relaxation behaviour observed in the low frequency region. This inevitable crossover to standard low frequency relaxation determines the observed crossover between *G*′(*ω*) and *G*′′(*ω*) at 28 °C.

### 3.3. Viscosities

**Fig. 6:**
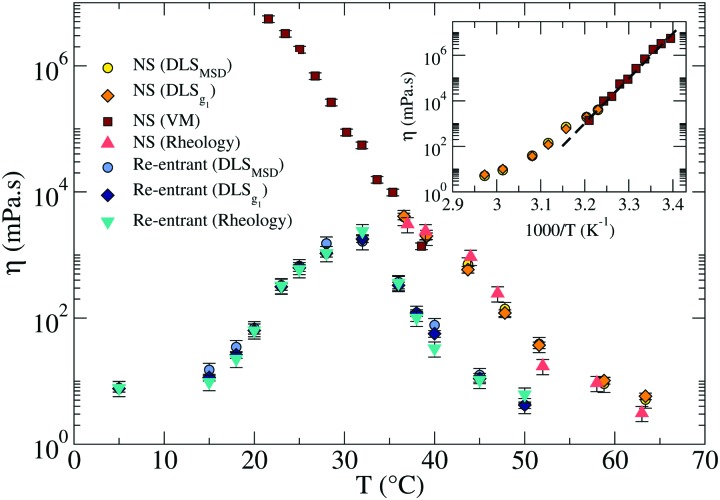
Comparison of the viscosities *η* in the RG (light and dark blue, and turquoise symbols) and in the NS (red, dark and light orange, and yellow symbols) systems. The inset shows the Arrhenius plot for the NS system in the gel region (at *T* below the melting temperature). The dashed line represents the best fitting with an exponential Arrhenius-like function with an activation energy *E*_A_ ≈ 105 kcal mol^–1^. The error bars are calculated as explained in Sections III and V of the ESI.†

In this subsection we complement the passive microrheology data by computing *η* for both the NS and the RG systems, using DLS and PTM techniques to measure the motion of tracer particles immersed in the sample. From the DLS data we extract *η* in two different ways. In the first method we evaluate the time *t*_1/*e*_ at which *g*_1_(*t*_1/*e*_) = 1/*e*, with *e* ≡ 2.7182, (which for a pure diffusive process coincides with (*Dq*^2^)^–1^) and convert it to *η* according to the Stokes–Einstein relation1
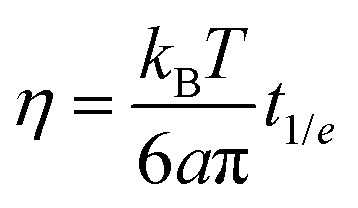
In the second method we extract the long time limit of the MSD (see Fig. 2(c) and 4(c, d)) calculated after CONTIN regularization to evaluate the diffusion coefficient. The two methods provide comparable results and the corresponding *η* are reported in Fig. 6.

In addition, it is possible to estimate the bulk viscositiy *η* directly from the rheological data as the limit for *ω* → 0 of *G*′′(*ω*)/*ω*. As shown in Fig. 6, the reported values by the three methods are all consistent.

Beside providing a set of independent measurements to validate the *η* values estimated from DLS, the PTM technique gives access to a different time window compared to DLS and allows us to extend the range over which *η* measurements are possible by a further three orders of magnitude.

**Fig. 7:**
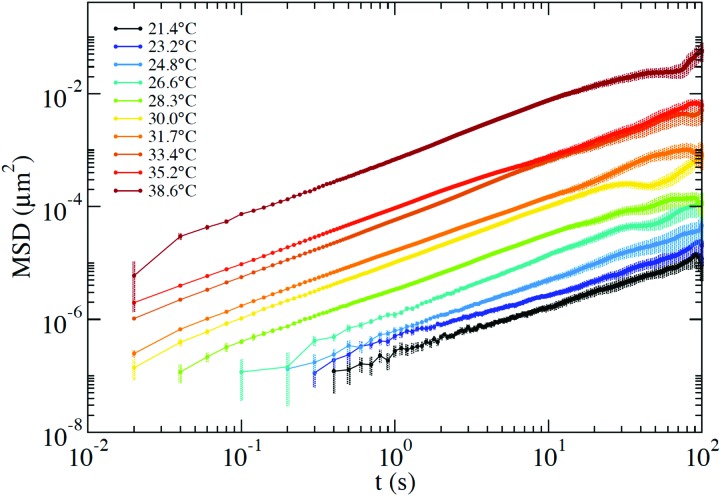
MSD measured *via* the PTM technique at *T* ranging from 21.4 °C up to 38.6 °C. The time-independent offset, which is affected by the finite exposure time and by tracking artefacts, has been subtracted from the curves. The error bars are computed as detailed in Section III of the ESI.†

From the PTM data we extract the positions over time of two tracer particles to compute the interparticle displacement, which undergoes a diffusive motion that is not affected by systematic errors associated with any source of global drift in the optical set-up. As discussed in detail in Section III of the ESI,† the selected exposure time does not allow us to quantify the amplitude of the short-time vibrational motion of the probe that would result in a positive *t* = 0 intercept of the MSD. In addition, the finite exposure time, as well as the tracking precision, introduces artefacts that make unphysical any interpretation of the intercept values.^47^ These values have been subtracted in the MSD presented in Fig. 7. Nevertheless, all this does not affect the evaluation of the slope of the MSD *vs.* time and thus of the diffusion coefficient, allowing us to compute *η via* the Stokes–Einstein relation.

Around *T* ≈ 38 °C, where both DLS and PTM measurements are possible, the agreement between DLS and PTM is highly satisfactory. On further cooling, *η* progressively increases as a result of the longer lifetime of the interparticle bonds. As expected for a strong network-forming fluid,^48^ at low *T*, *η* follows an Arrhenius behavior with an activation energy *E*_A_ ≈ 105 kcal mol^–1^ which reflects the enthalpy of the inter particle bonds (44.6 kcal mol^–1^ (ref. 49)).

For the RG, *η* was evaluated *via* DLS, also shown in Fig. 6. The *T*-dependence of *η* shows a clear maximum at intermediate *T*. Consistently with the behavior displayed by the linear viscoelastic moduli, *η* first increases on cooling when the network progressively forms and then decreases again when the competitor sequences start binding, capping the NS sticky ends until the point at which, at very low *T*, the network is completely disentangled and fluid-like behavior is recovered. At this point a *η* comparable to the one reported at 50 °C is again observed, which is only few times higher than the solvent *η*. In the case of the RG, an accurate estimate of the activation energy is not possible, since the system is designed to evolve back to a fluid at low *T*. In the region of *T* where *η* increases, the slope of ln *η vs. T*^–1^ progressively changes with *T* preventing an accurate estimate of the activation energy.

## 4. Conclusions

We have presented experimental characterisation of two DNA hydrogels resulting from the thermoreversible association of DNA-made nanoparticles with functionality four. The possibility to exploit base-pair selectivity and the self-assembly properties of DNA sequences of different length permitted us to tune the viscoelastic properties as desired, providing significant design flexibility compared to tetravalent star polymers.^50^–^52^ Specifically, we have compared a system that gels on cooling (the NS system), with a system that gels both on cooling, when starting from high *T*, and on heating, when starting from low *T* (the RG system). We can envision that the mechanical properties of all-DNA hydrogels can be controlled by the functionality of the NSs, and by their concentration and stoichiometry. This may offer control over the viscoelastic properties of this class of materials.

The frequency-dependent viscoelastic moduli measured with DLS passive microrheology revealed the different viscoelastic properties of the two systems as a function of *T*. The data showed the crossover from a fluid to a gel for the NS system *via* a percolation transition, and a multiple sequence of transitions from a fluid to a percolating system to an elastic gel to a percolating system and to a fluid again on cooling, for the RG system. Similarly, *η* monotonically increased on cooling in the NS system while it first increased and then decreased again in the RG system. The combination of DLS and PTM techniques allowed us to explore more than six orders of magnitude in *η*.

In conclusion, we provide evidence of the possibility of measuring material properties in hydrogels built by controlled association of DNA-made particles, with specified functionality and binding sequences. The comparison between the NS and RG systems highlights both the flexibility offered by the design of the DNA sequences and the possibility to tune the mechanical response of these biocompatible all-DNA materials with minor changes in the DNA sequences.^53^ The biocompatibility and thermoreversible character of these materials opens the possibility for a large number of applications related to the biomedicine and drug delivery fields in which fine control of the materials' mechanical properties is required.

## Conflicts of interest

The authors declare no competing financial interests.

## Supplementary Material

Supplementary information
