# Supplementary material for: Microrheology of DNA hydrogel gelling and melting on cooling
Source: Soft Matter. 2018 Jun 19;14(31):6431–8. doi: 10.1039/c8sm00751a (PMC6295875; doi:10.1039/c8sm00751a)
Supplement: Supplementary file 1 [file SM-014-C8SM00751A-s001.pdf]

Electronic Supplementary Material (ESI) for Soft Matter.
This journal is © The Royal Society of Chemistry 2018

SUPPORTING INFORMATION: Microrheology of DNA Hydrogels Gelling and Melting on Cooling

I. POLYSTYRENE (PS) TRACERS IN WATER

To evaluate the optimal colloidal (tracer) volume fraction (ϕ), and to check the suitability of the instrumental set up we performed a preliminar study with streptavidin coated PS colloids of nominal diameter 530 nm at different values of ϕ in a buffer of H_2O and 100 mM of NaCl.

The typical scattering volume in the DLS apparatus is estimated to be of the order of $\sim 10^{-6}$ cm^3, assuming that the laser beam is focused to 200 μm and the scattering angle is fixed to 90°. It is generally considered that 300 scatterers per scattering volume are required in order to guarantee gaussian statistics of the scattered field and to properly apply the Siegert relationship[1]. This corresponds to a lower limit for the number density of roughly $\sim 10^8$ particles/cm^3 or to a lower limit for the volume fraction $\phi \sim 10^{-5}$.

Fig. 1 shows the $g_1(t)$ autocorrelation function measured at $T = 20$°C and wave vector $q = 0.0186$ nm^{-1} for three different values of ϕ. All curves are rather well described by a single exponential decay (black solid lines).

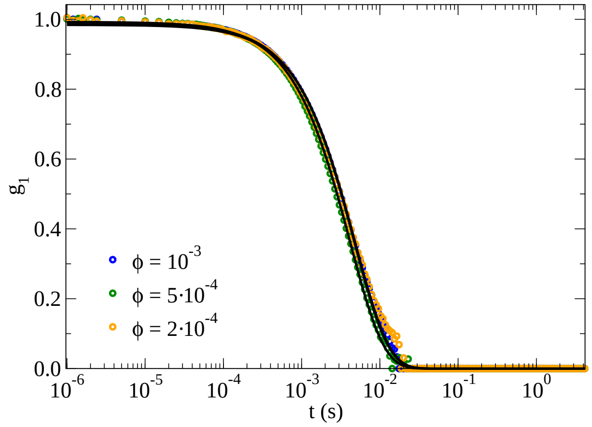

FIG. 1. Autocorrelation function $g_1(t, q)$ for PS colloids at three different volume fractions ϕ in a buffer of water at 100 mM NaCl. The black solid lines correspond to the best fitting curves of a single exponential function.

A proper passive microrheology experiments requires that the probe particles do not interact, such that the measured scattering function is dominated by the self component. It is normally assumed that hard sphere particles do not interact when the distance between them is larger than 20 times their diameter[1]. This factor 20 leads to a maximum $\phi \sim 6 \cdot 10^{-3}$. Indeed, with the aim to minimise particle interactions and to guarantee the validity of this approximation we select a volume fraction one order of magnitude lower ($\phi = 3.4 \cdot 10^{-4}$). Under this condition, $g_1(\tau) = exp(-q^2 < r^2(\tau) > /6)$, where $< r^2(\tau) >$ is the mean square displacement of the probe particle. In addition, at this value, the scattering intensity from the colloidal particles is observed to be more than twenty times larger than the one of a DNA hydrogel sample. As a further check we evaluated the viscosity (η) of water at different Ts ranging from 15°C up to 52°C. The DLS results and the corresponding viscosities are shown in Fig. 2 together with the theoretical values from the NIST database[2]. Finally, from the single exponential fittings of the autocorrelation functions presented in Fig. 2(a) it is possible to estimate the hydrodynamic radius of the colloidal tracers. The average of the measurements, using as reference η of water at the corresponding Ts, it is estimated in $\sim 268 \pm 5$ nm.

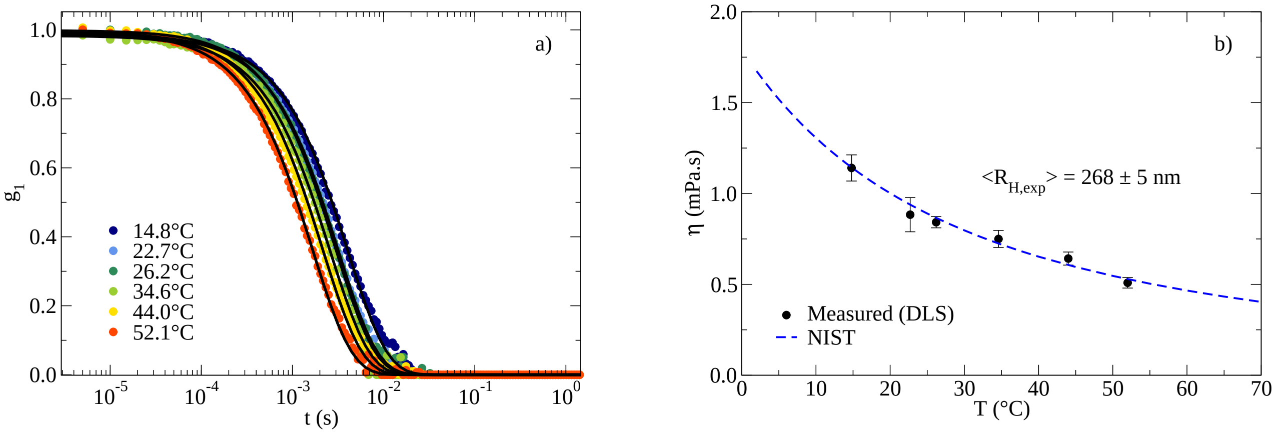

FIG. 2. (a) ACF $g_1(t,q)$ at different Ts for PS colloids at a fixed volume fraction $\phi = 3.4\ 10^{-4}$ in a buffer of water at 100 mM NaCl. The black solid lines correspond to the best fitting curves of a single exponential function. (b) Black circles indicate the measured experimental η at different Ts calculated from the data shown in Fig. 2. The dashed blue line represent the theoretical values for η of water from the NIST database. The tracer hydrodynamic radius is estimated in $\sim 268 \pm 5$ nm.

II. DNA SEQUENCES

The tetrafunctional DNA particles of the first hydrogels system were prepared by diluting equimolar concentrations of the sequences $\mathbf{NS}_1$-$\mathbf{NS}_4$

$\mathbf{NS}_1$. 5′-CTACTATGGCGGGTGATAAAAACGGGAAGAGCATGCCCATCCA**CGATCG**-3′
$\mathbf{NS}_2$. 5′-GGATGGGCATGCTCTTCCCGAACTCAACTGCCTGGTGATACGA**CGATCG**-3′
$\mathbf{NS}_3$. 5′-CGTATCACCAGGCAGTTGAGAACATGCGAGGGTCCAATACCGA**CGATCG**-3′
$\mathbf{NS}_4$. 5′-CGGTATTGGACCCTCGCATGAATTTATCACCCGCCATAGTAGA**CGATCG**-3′

where sequences with the same colour indicate complementary strands.

In the reentrant-gel system, four sequences ($A_1 - A_4$) are needed to form the tetrafunctional nanostar structures and two single stranded sequences ($B_1 - B_2$) to form the so-called *competitors*:

$\mathbf{A}_1$. 5'-CTACTATGGCGGGTGATAAAAACGGGAAGAGCATGCCCATCCATGAGCGTACGCAAT-3'
$\mathbf{A}_2$. 5'-GGATGGGCATGCTCTTCCCGAACTCAACTGCCTGGTGATACGATGAGCGTACGCAAT-3'
$\mathbf{A}_3$. 5'-CGTATCACCAGGCAGTTGAGAACATGCGAGGGTCCAATACCGATGAGCGTACGCAAT-3'
$\mathbf{A}_4$. 5'-CGGTATTGGACCCTCGCATGAATTTATCACCCGCCATAGTAGATGAGCGTACGCAAT-3'
$\mathbf{B}_1$. 5'-ATTGCG-3'
$\mathbf{B}_2$. 5'-CGCTCA-3'

Here the nucleotides involved in the formation of the arms of the tetravalent nanostar are reported in blue, followed by a 12-long sticky-end sequence. Competitor particles are designed to be complementary to different parts of the nanostar sticky overhangs, being able to cap the nanostar sticky-ends at low T. The breaking of the inter nanostar bonds results in the melting on cooling of the gel network. The characteristic re-entrant behaviour introduced by the competition between the configurations stabilised by energy and those stabilised by entropy is discussed in more detail in Ref. 3.

III. EVALUATION OF THE VISCOSITY FROM THE MSD USING PARTICLE TRACKING MICROSCOPY (PTM)

Calling (x^a, y^a) and (x^b, y^b) the discretized 2D trajectory of two tracked PS microspheres (whose relative distance is always larger than 7 μm) we compute the MSDs along the x and y axis as:

$$\mathrm{MSD}_x(\Delta t_i) = \sum_j \left(x(t_j + \Delta t_i) - x(t_j)\right)^2 \quad (1)$$

$$\mathrm{MSD}_y(\Delta t_i) = \sum_j \left(y(t_j + \Delta t_i) - y(t_j)\right)^2 \quad (2)$$

where $x(t) \equiv (x^a(t) - x^b(t))/\sqrt{2}$ and $y(t) \equiv (y^a(t) - y^b(t))/\sqrt{2})$. The sum runs over all the possible time origins for which $t_j + \Delta t_i$ does not exceed the observation time. To properly cover the diffusive part of the trajectory we use a frame rate between 10 and 50 frames per second, with a exposure time T_e of 10 ms. This T_e value allows us to properly measure η but it prevents us from exploring the viscoelastic behaviour of the medium. Figures 3(a) and 3(b) show the measured MSD_x and MSD_y at two different temperatures, 21°C and 39°C, respectively.

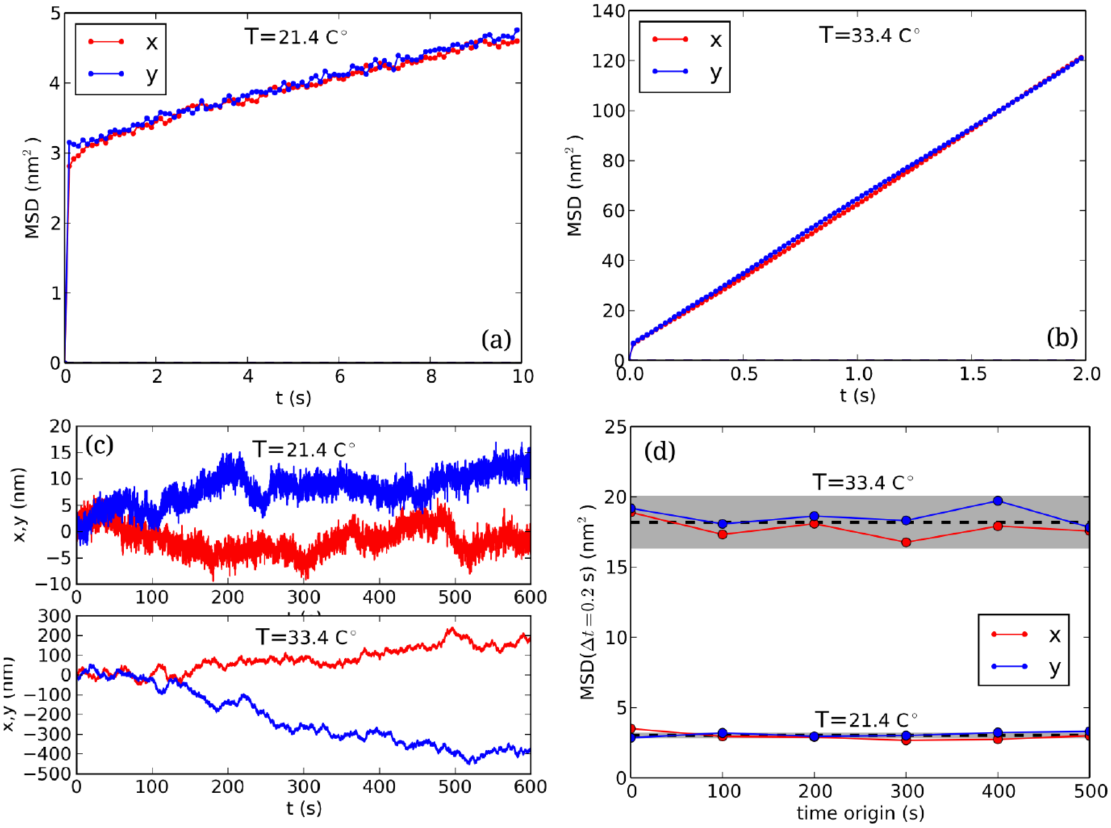

FIG. 3. MSD for (a) $T = 21$°C and (b) $T = 39$°C in independent directions x (black circles) and y (green circles). (c) x and y trajectories for two temperatures T = 21.4°C (top) and T = 33.4°C (bottom). (d) MSD at T = 21.4°C (top) and T = 33.4°C for a fixed Δt = 0.2 s. The grey shaded area indicates the expected fluctuations.

Both independent directions provide the same MSD. The measured MSDs grow linearly with time, showing a finite intercept at $t = 0$. Both MSD_x and MSD_y can be accurately fitted with a linear function $MSD = 2Dt + q$, where D and q are the fitting parameters.

Given the large distance between the two colloids, hydrodynamic interactions can be neglected. We thus assume the microspheres' Brownian displacements to be uncorrelated. In this case, eq. 1, for both x and y directions, results in a MSD growing at long times as $(D^a + D^b)t$ where D^a and D^b are the diffusion coefficients of the two microspheres. Finally, considering an equal size of the microspheres' (within a 5% uncertainty coming from colloids' radii polydispersity), we identify $D^a = D^b = D$. Having access to two independent measurements of the MSD, respectively along x and y directions, the precision of the fitting procedure can be estimated. Defining D_x and D_y as the values corresponding to the fit of MSD_x and MSD_y, we estimate the relative error as the average value of $2|D_x - D_y|/(D_x + D_y)$ for all the investigated temperatures, resulting in a relative error of $\sim 10\%$. When computing η *via* the Stokes-Einstein relation, we also take into account the 5% uncertainty on the microspheres' radii, obtaining a final 12% precision.

We note that with the selected experimental set-up, the value of q does not provide a measure of the squared size of the typical cage confining the probe particle. Indeed, the value of q is affected by instrumental tracking artefacts due to the finite camera exposure time, since the measured position of a colloid corresponds to the temporal average

of the real trajectory during T_e. Following Ref. 4, the experimentally observed mean square displacement MSD_e at time Δt is related to the real MSD by an averaging procedure over the exposure time T_e (that we assume larger than the time the particle spends in the cage T_{cage})

$$\text{MSD}_e(\Delta t) = \frac{1}{T_e^2}\int_0^{T_e} dt' \left[\text{MSD}(\Delta t + t') + \text{MSD}(\Delta t - t') - 2\text{MSD}(t')\right](T_e - t') \tag{3}$$

A detailed computation of MSD_e is described in Ref. 4. Here, we will provide a back of the envelope evaluation of MSD_e for the case in which Δt is larger than the caging time ($\Delta t >> T_e > T_{cage}$). In Equation 3 we approximate the (one-dimensional) MSD with the linear function

$$MSD(t) \approx 2Dt + \xi^2. \tag{4}$$

Correspondingly, we substitute $\text{MSD}(\Delta t \pm t') = 2D(\Delta t \pm t') + \xi^2$ and $\text{MSD}(t') \approx \xi^2$ where we assumed a relaxation time in the cage T_{cage} shorter than T_e. After integration we find $\text{MSD}_e(\Delta t) = 2D\Delta t$ leading to $q \approx 0$. Therefore, a finite exposure time $T_e > T_{cage}$, leads to an error in the intercept of the order of the of the squared cage size. Additionally, by taking into account the tracking error associated to the noise and pixelation of the camera (~ 3 nm) the experimental value q for the intercept becomes unusable. For this reason, the fitted intercept value from the MSD curves displayed in Fig. 7 of the main text has been removed.

Errors on the MSD shown in Fig. 7 have been estimated by dividing the 10 minutes long trajectories into N_{sub} subtrajectories of length $T_{sub} = 100$ s. For each subtrajectory we compute the MSD along a coordinate (x or y) that we indicate with $\text{MSD}_i(\Delta\text{t})$, where $i = 1, 2, \ldots, 6$ accounts for the subset of trajectories. For each time Δt we compute the mean $\text{MSD} = {N_{sub}}^{-1}\sum_i \text{MSD}_i(\Delta\text{t})$. The standard error of the mean MSD at a selected lag time Δt is taken as the standard deviation of $\text{MSD}_i(\Delta\text{t})/N_{sub}^{1/2}$.

In a similar way, in order to verify the stationarity of the diffusion process, again for each temperature, we plot the $\text{MSD}_i(\Delta\text{t})$ at a fixed Δt (see Fig. 3). MSD_i fluctuates around its mean value within the expected standard deviation, confirming the stationarity of the diffusion processes.

IV. THEORETICAL BACKGROUND: DLS MICRORHEOLOGY

The $g_1(t)$ is related to the mean square displacement (MSD) of the colloidal tracer particle $< \Delta r^2(\tau) >$ by

$$g_1(\tau, q) = g_1(0)\exp\left(\frac{-q^2 < \Delta r^2(\tau) >}{6}\right) \tag{5}$$

where the wave vector q is defined as

$$q = \frac{4\pi n}{\lambda}\sin\left(\frac{\theta}{2}\right) \tag{6}$$

and n stands for the refractive index of the medium, λ is the laser wavelength and θ is the scattering angle.

DLS microrheology relies on the thermal energy $k_B T$ associated to the tracer colloids. According to the generalized Stokes-Einstein (GSER) equation[5], defined in a similar way to the Stokes-Einstein equation but as a function of Laplace transformed quantities, it is possible to relate the diffusion coefficient (D) to the viscosity (η) of the material as

$$\tilde{D}(s) = \frac{k_B T}{6\pi a s \tilde{\eta}(s)} \tag{7}$$

where $\tilde{D}(s)$ and $\tilde{\eta}(s)$ are the diffusion coefficient and the viscosity as a function of the Laplace frequency s and a is the tracer particle radius. From eq. 7, and assuming that inertial effects are negligible at the experimentally accessible ω, the Laplace transformed of the complex modulus $\tilde{G}(s)$ can be expressed as[6]

$$\tilde{G}(s) = s\tilde{\eta}(s) = \frac{s}{\pi a}\left[\frac{k_B T}{s^2 < \Delta\tilde{r}^2(s) >}\right] \tag{8}$$

where $< \Delta\tilde{r}^2(s) >$ represents the Laplace transform of the measured MSD. Then, the linear viscoelastic moduli as a function of the frequency ω, $G'(\omega)$ and $G''(\omega)$, can be obtained according to the frequency transformation $s = i\omega$ and to the relation $G^*(\omega) = G'(\omega) + iG''(\omega)$[6–8]. Here, $G'(\omega)$ and $G''(\omega)$ represent the real and the imaginary part of the complex modulus $G^*(\omega)$ which are related to the elasticity and the viscosity of the material, respectively. As a result

$$G^*(\omega) = \frac{k_B T}{\pi a i \omega \mathfrak{F}\{< \Delta r^2(\tau) >\}} \tag{9}$$

where $\mathfrak{F}\{< \Delta r^2(\tau) >\}$ is the one side Fourier transform of the MSD.

A convenient method to avoid spurious fluctuations in the Laplace and Fourier transforms has been developed in Ref. [7, 4, 9] and often applied to soft-matter systems[10]. $< \Delta r^2(\tau) >$ is expanded locally around $\tau = 1/\omega$ assuming

$$< \Delta r^2(\tau) >=< \Delta r^2(1/\omega) > \left(\frac{\tau}{1/\omega}\right)^{\alpha(\omega)} \tag{10}$$

where $\alpha(\omega)$ is estimated as the local slope of the MSD logarithmic time derivative

$$\alpha(\omega) = |[\partial ln < \Delta r^2(\tau) > /\partial ln \tau]|_{\tau=1/\omega}. \tag{11}$$

In purely viscous media $\alpha(\omega)$ takes a value of 1, while if the particle is completely arrested in an elastic medium the MSD slope would be 0. Therefore, in viscoelastic materials, $\alpha(\omega)$ is expected to range within these two cases, $0 < \alpha(\omega) < 1$. From eq. 10 and eq. 11, the GSER in the Fourier space can be expressed as

$$G'(\omega) = |G^*(\omega)| \cos[\pi\alpha(\omega)/2] \tag{12}$$

$$G''(\omega) = |G^*(\omega)| \sin[\pi\alpha(\omega)/2] \tag{13}$$

where

$$|G^*(\omega)| = \frac{k_B T}{\pi a < \Delta r^2(1/\omega) > \Gamma[1 + \alpha(\omega)]} \tag{14}$$

being Γ the gamma function. Two limits are worth discussing. If the MSD is time-independent (complete caging, $< \Delta r^2(\tau) >= \xi_0^2$) then $\alpha(\omega) = 0$ and

$$|G^*(\omega)| = \frac{k_B T}{\pi a \xi_0^2}, \qquad G'(\omega) = |G^*(\omega)| \qquad G''(\omega) = 0 \tag{15}$$

If instead the MSD is scale invariant, $< \Delta r^2(\tau) >= \xi_0^2 \left(\frac{t}{t_0}\right)^{\beta}$, as expected for simple diffusion ($\beta = 1$) and close to percolation ($\beta \neq 1$) then $\alpha(\omega) = \beta$ is frequency independent and

$$|G^*(\omega)| = \frac{k_B T}{\pi a} \frac{1}{\xi_0^2 \left(\frac{1}{\omega t_0}\right)^{\beta} \Gamma[\beta + 1]} \tag{16}$$

$$G'(\omega) = |G^*(\omega)| \cos(\beta\pi/2) \qquad G''(\omega) = |G^*(\omega)| \sin(\beta\pi/2)$$

Hence G' and G'' are parallel power-law in ω. If $\beta = 0.5$ $G'(\omega) = G''(\omega)$.

Finally, η can be calculated equivalently as the $\lim_{\omega\to 0} G''(\omega)/\omega$ or from the long time limit of the MSD, exploiting the Stokes-Einstein relation[11]

$$< \Delta r^2(\tau) >= 6D\tau = \frac{k_B T}{\pi \eta a}\tau \tag{17}$$

V. STATISTICAL ERRORS FROM DLS MEASUREMENTS

In DLS microrheology both the mean-square displacement (MSD) and the viscoelastic moduli (G' and G'') are directly calculated from the intensity autocorrelation functions. The latter are affected by statistical errors due to the finite time of the measurements (t_M) that in consequence are propagated to the MSD and viscoelastic moduli.

Following the work of Degiorgio and Lastovka[12] the statistical error of the intensity autocorrelation function $g_2(\tau)$ can be approximated by

$$\Delta g_2(\tau) \approx 6\sqrt{\frac{\tau}{t_M}}, \tag{18}$$

where τ corresponds to the correlation time.

The field autocorrelation function $g_1(\tau)$ is related to $g_2(\tau)$ through the Siegert relation $g_1(\tau) = \sqrt{(g_2(\tau)-1)/C} \approx \sqrt{(g_2(\tau)-1)}$, where $C \sim 1$ is a correction factor that accounts for the quality of the alignment and the geometry of the optical experimental set-up.

The statistical error of $g_1(\tau)$ can be then calculated as:

$$\frac{\Delta g_1}{g_1} = \frac{1}{g_1}\frac{\partial g_1}{\partial g_2}\Delta g_2 \approx \frac{6}{2g_1\sqrt{g_2-1}}\sqrt{\frac{\tau}{t_M}} \approx \frac{3}{g_1^2}\sqrt{\frac{\tau}{t_M}}$$

therefore,

$$\Delta g_1 \approx \frac{3}{g_1}\sqrt{\frac{\tau}{t_M}} \tag{19}$$

From eq. 5 it is possible to derive the relative error associated to the MSD and from eq. 14 we will assume that $\Delta G^*/G^* \approx \Delta MSD/MSD$,

$$\frac{\Delta G^*}{G^*} \approx \frac{\Delta MSD}{MSD} \approx \frac{1}{MSD}\frac{\partial MSD}{\partial g_1}\Delta g_1 \approx \frac{q^2}{6ln(g_1)}\frac{6}{q^2 g_1}\frac{3}{g_1}\sqrt{\frac{\tau}{t_M}} \approx \frac{3}{g_1^2 ln(g_1)}\sqrt{\frac{\tau}{t_M}} \tag{20}$$

[1] Brookhaven Instr. Coorp., *Instruction Manual for BI-9000AT Digital Autocorrelator*, Vol. Ver. 1.0. (9kDLSW Manual, 1998).
[2] The National Institute of Standards and Technology (NIST), "Data From NIST Standard Reference Database 69: NIST Chemistry WebBook," (Last Revised 2018).
[3] F. Bomboi, F. Romano, M. Leo, J. Fernandez-Castanon, R. Cerbino, T. Bellini, F. Bordi, P. Filetici, and F. Sciortino, "Re-Entrant DNA Gels," Nat. Commun. **7** (2016).
[4] T. Mason, K. Ganesan, J. Van Zanten, D. Wirtz, and S. Kuo, "Particle Tracking Microrheology of Complex Fluids," Phys. Rev. Lett. **79**, 3282 (1997).
[5] P. M. Chaikin and T. C. Lubensky, *Principles of Condensed Matter Physics* (Cambridge university press, 2000).
[6] T. G. Mason and D. Weitz, "Optical measurements of frequency-dependent linear viscoelastic moduli of complex fluids," Phys. Rev. Lett. **74**, 1250 (1995).
[7] T. G. Mason, "Estimating the viscoelastic moduli of complex fluids using the generalized stokes–einstein equation," Rheol. Acta **39**, 371–378 (2000).
[8] A. J. Levine and T. Lubensky, "One-and two-particle microrheology," Phys. Rev. Lett. **85**, 1774 (2000).
[9] B. Dasgupta, J. Crocker, S. Tee, and D. Weitz, "Microrheology of polyethylene oxide using dws and single scattering," in *APS Meeting Abstracts* (2000).
[10] J. Liu, V. Boyko, Z. Yi, and Y. Men, "Temperature-dependent gelation process in colloidal dispersions by diffusing wave spectroscopy," Langmuir **29**, 14044–14049 (2013).
[11] F. Reif, *Statistical Thermal Physics* (Mcgraw-Hill Kogakusha, 1965).
[12] V. Degiorgio and J. Lastovka, "Intensity-correlation spectroscopy," Physs Rev. A **4**, 2033 (1971).
